# The impact of the genome-wide supported variant in the cyclin M2 gene on gray matter morphology in schizophrenia

**DOI:** 10.1186/1744-9081-9-40

**Published:** 2013-10-25

**Authors:** Kazutaka Ohi, Ryota Hashimoto, Hidenaga Yamamori, Yuka Yasuda, Michiko Fujimoto, Satomi Umeda-Yano, Masaki Fukunaga, Yoshiyuki Watanabe, Masao Iwase, Hiroaki Kazui, Masatoshi Takeda

**Affiliations:** 1 Department of Psychiatry, Osaka University Graduate School of Medicine, Osaka, Japan; 2 National Hospital Organization, Yamato Mental-Medical Center, Nara, Japan; 3 Core Research for Evolutionary Science and Technology of the Japan Science and Technology Agency, Saitama, Japan; 4 Molecular Research Center for Children’s Mental Development, United Graduate School of Child Development, Osaka University, Osaka, Japan; 5 Department of Molecular Neuropsychiatry, Osaka University Graduate School of Medicine, Osaka, Japan; 6 Biofunctional Imaging, Immunology Frontier Research Center, Osaka University, Osaka, Japan; 7 Department of Radiology, Osaka University Graduate School of Medicine, Osaka, Japan

**Keywords:** Schizophrenia, Genome-wide association study, Voxel-based morphometry, Cyclin M2 (*CNNM2*), Inferior frontal gyrus

## Abstract

**Background:**

Genome-wide significant associations of schizophrenia with eight SNPs in the *CNNM2*, *MIR137*, *PCGEM1*, *TRIM26*, *CSMD1*, *MMP16*, *NT5C2* and *CCDC68* genes have been identified in a recent mega-analysis of genome-wide association studies. To date, the role of these SNPs on gray matter (GM) volumes remains unclear.

**Methods:**

After performing quality control for minor-allele frequency > 5% using a JPT HapMap sample and our sample, a genotyping call rate > 95% and Hardy-Weinberg equilibrium testing (*p* > 0.01), five of eight SNPs were eligible for analysis. We used a comprehensive voxel-based morphometry (VBM) technique to investigate the effects of these five SNPs on GM volumes between major-allele homozygotes and minor-allele carriers in Japanese patients with schizophrenia (*n* = 173) and healthy subjects (*n* = 449).

**Results:**

The rs7914558 risk variant at *CNNM2* was associated with voxel-based GM volumes in the bilateral inferior frontal gyri (right *T* = 4.96, *p* = 0.0088, left *T* = 4.66, *p* = 0.031). These peak voxels, which were affected by the variant, existed in the orbital region of the inferior frontal gyri. Individuals with the risk G/G genotype of rs7914558 had smaller GM volumes in the bilateral inferior frontal gyri than carriers of the non-risk A-allele. Although several effects of the genotype and the genotype-diagnosis interaction of other SNPs on GM volumes were observed in the exploratory VBM analyses, these effects did not remain after the *FWE-*correction for multiple tests (*p* > 0.05).

**Conclusions:**

Our findings suggest that the genetic variant in the *CNNM2* gene could be implicated in the pathogenesis of schizophrenia through the GM volumetric vulnerability of the orbital regions in the inferior frontal gyri.

## Background

Schizophrenia is a common and complex psychiatric disorder with a lifetime risk of approximately 1%. This disorder has a strong genetic component; indeed, the estimated heritability is 81% [1]. Multiple genetic variants that have a small effect have been implicated in the pathogenesis of schizophrenia [2]. A genome-wide association study (GWAS) of single-nucleotide polymorphisms (SNPs) that accesses tens of thousands of DNA samples from patients and controls can be a powerful tool for identifying common risk factors for complex diseases, such as schizophrenia. To date, GWASs on schizophrenia have identified several genome-wide significant associated variants located in the zinc finger protein 804A (*ZNF804A*), neurogranin (*NRGN*), transcription factor 4 (*TCF4*) genes and a major histocompatibility complex (MHC) region [3,4]. Subsequently, the influences of these SNPs in the genes on brain function and structure have been reported [5]. We have found that the genome-wide supported variant of the *NRGN* gene is associated with the brain morphology of the anterior cingulate cortex in patients with schizophrenia [6].

Recently, a combining analysis of a mega-analysis of GWAS data from 17 separate studies (9,394 cases and 12,462 controls) and the replication data (8,442 cases and 21,397 controls) of European ancestry have found genome-wide significant associations of schizophrenia with eight SNPs [rs7914558 (cyclin M2; *CNNM2*), rs1625579 (microRNA 137, *MIR137*), rs17662626 (PCGEM1, prostate-specific transcript, *PCGEM1*), rs2021722 (tripartite motif containing 26, *TRIM26*), rs10503253 (CUB and Sushi multiple domains 1, *CSMD1*), rs7004633 (matrix metallopeptidase 16, *MMP16*), rs11191580 (5′-nucleotidase, cytosolic II, *NT5C2*) and rs12966547 (coiled-coil domain containing 68, *CCDC68*)] from the five new (1p21.3, 2q32.3, 8p23.2, 8q21.3 and 10q24.32-q24.33) and two previously reported (6p21.32-p22.1 and 18q21.2) loci [7]. In studies exploring brain activation during a sentence completion task, individuals without the risk allele of the *MIR137* genotype had significantly greater activation in the posterior right medial frontal gyrus than at-risk individuals [8]. To our knowledge, however, no study has investigated the effects of these SNPs on gray matter (GM) volumes.

Many attempts have been made to minimize the clinical and genetic heterogeneity in studies of schizophrenia. One strategy for gene discovery uses neurobiological quantitative traits (QT) as intermediate phenotypes that reflect the underlying genetic vulnerability better than diagnostic categorization, such as schizophrenia [9,10]. This strategy has the potential to reduce clinical and genetic heterogeneity [11]. Structural GM volumes indicate substantial heritability rates ranging from moderate (40–70%) to high (70–95%) in the frontal and temporal brain regions [12,13]. Mata analyses of brain morphological studies in individuals with first-episode schizophrenia and neuroleptic naive schizophrenia as well as chronic patients with schizophrenia revealed reduction of GM volume in frontal, striato-limbic and temporal regions were present in the early stage of schizophrenia and were unrelated to the effects of neuroleptic treatment, chronicity and duration of illness [14-16]. Some studies have shown that abnormalities in GM volumes are intermediate phenotypes that bridge the gap between the genotype and diagnostic categorization [11,17]. Characterizing the functional effects of novel and poorly understood genetic variants on the intermediate phenotypes provides important insights into the neural mechanisms by which the variants increase the risk for schizophrenia [18]. Our research group has had a long-standing interest in the effects of genetic variants (i.e., *COMT*, *DISC1*, *PACAP*, *BDNF*, *APOE*, *AKT1* and *NRGN*) on brain structure in psychiatric disorders [6,19-24]. In this study, we examined the impact of the genome-wide supported variants on the GM volumes of patients with schizophrenia and healthy subjects. Using a comprehensive voxel-based morphometry (VBM) technique, we tested the hypothesis that these risk variants would be associated with GM volumes.

## Material and methods

### Subjects

VBM analyses were conducted on 173 patients with schizophrenia (59.0% males, 102 males and 71 females, mean age ± SD, range 36.0 ± 12.3 years) and 449 healthy subjects (47.7% males, 214 males and 235 females, mean age ± SD, range 35.4 ± 12.8 years). All subjects were biologically unrelated within the second-degree of relationship and were of Japanese descent [25,26]. The subjects were excluded if they had neurological or medical conditions that could potentially affect the central nervous system, such as atypical headache, head trauma with loss of consciousness, chronic lung disease, kidney disease, chronic hepatic disease, thyroid disease, active cancer, cerebrovascular disease, epilepsy, seizures, substance-related disorders or mental retardation. Patients were recruited from the Osaka University Hospital. Each patient with schizophrenia had been diagnosed by at least two trained psychiatrists according to the criteria from the *Diagnostic and Statistical Manual of Mental Disorders, Fourth Edition* (DSM-IV) based on the Structured Clinical Interview for DSM-IV (SCID). Controls were recruited through local advertisements at Osaka University. Psychiatrically, medically and neurologically, the healthy subjects were evaluated using the non-patient version of the SCID to exclude individuals who had current or past contact with psychiatric services or who had received psychiatric medication. Current symptoms of schizophrenia were evaluated using the positive and negative syndrome scale (PANSS) [27]. Mean age and handedness did not differ significantly between the study group and the controls (*p* > 0.38), while the female ratio, years of education, estimated premorbid intelligence quotient (IQ) and total gray matter volumes were significantly lower in the patients with schizophrenia compared to the controls (*p* < 0.016) (Additional file 1: Table S1). All participants provided written informed consent after the study procedures had been fully explained. This study was performed in accordance with the World Medical Association’s Declaration of Helsinki and was approved by the Research Ethical Committee of Osaka University.

### SNP selection and SNP genotyping

We selected eight SNPs, rs7914558, rs1625579, rs17662626, rs2021722, rs10503253, rs7004633, rs11191580 and rs12966547, from a previous mega analysis of GWASs [7]. Of these eight SNPs, rs17662626 in the *PCGEM1* gene (2q32.3) was not polymorphic in the samples obtained from the HapMap Japanese in Tokyo (JPT) project. Because the other seven SNPs were common genetic variants in the HapMap JPT samples (minor allele frequency > 5%), we focused on these SNPs. Venous blood was collected from the subjects, and genomic DNA was extracted from whole blood according to standard procedures. These SNPs were genotyped using the TaqMan 5′-exonuclease allelic discrimination assay (Assay ID: rs7914558: C__31978821_10, rs1625579: C___8946584_20, rs2021722: C__11690541_10, rs10503253: C___1503810_20, rs7004633: C__29048976_10, rs11191580: C__31656012_10 and rs12966547: C____152930_10, Applied Biosystems, Foster City, CA, USA), as previously described [19,22]. Detailed information on the PCR conditions is available upon request. The genotyping call rates were 98.2% (rs7914558), 97.9% (rs1625579), 83.6% (rs2021722), 97.9% (rs10503253), 97.9% (rs7004633), 99.8% (rs11191580) and 97.6% (rs12966547). No deviation from the Hardy-Weinberg equilibrium (HWE) in the examined SNPs was detected in the patients or in controls (*p* > 0.01), with the exception of rs2021722. A significant deviation from HWE in the rs2021722 was found in both the patients (*p* = 1.02 × 10^-17^) and controls (*p =* 2.05 × 10^-36^) with a relative excess of CC homozygotes, TT homozygotes and undetermined subjects. According to the dbSNP database (National Center for Biotechnology Information), the SNP is shown as a tri-allelic variant with T/C/A. A number of genome-wide significant variants within MHC (6p21.32-p22.1), including rs2021722, have been identified [7]. However, the MHC region has been excluded from further analysis. Analyzing the region is difficult because of its high linkage disequilibrium (LD) and ethnic heterogeneity. Minor allele frequencies of rs1625579 were under 5% in our patients (3.2%) and controls (2.5%). Therefore, in this study, we excluded these SNPs rs2021722 and rs1625579 from the VBM analyses. Genotype and allele distributions for each SNP included in the VBM analyses between the patients with schizophrenia and the controls are shown in Table 1. All risk alleles were defined based on the previous GWAS [7]: rs7914558 (major G-allele), rs10503253 (minor A-allele), rs7004633 (minor G-allele), rs11191580 (major T-allele) and rs12966547 (minor G-allele). To increase the statistical power and decrease type I errors, homozygotes and the heterozygotes for the minor allele groups were combined and treated as minor-allele carriers. In this study, we contrasted GM volumes between minor allele carriers and major allele homozygotes.

**Table 1:** Genotype and allele distributions for each SNP between the patients with schizophrenia and healthy subjects

**SNP IDs**	**Gene**	**Chr**	**Risk**	**Genotype frequencies**						**Risk allele**		**Allelic**	**OR**
			**allele**	**+/+**	**+/-**	**-/-**	**+/+**	**+/-**	**-/-**	**frequencies**		***p*** **value**	**(95% CI)**
			**+/-**	**SCZ (*n*** **= 173)**			**CON (*n*** **= 449)**			**SCZ**	**CON**	**(*χ2*)**	
rs10503253	*CSMD1*	8p23.2	A/C	0.06	0.46	0.49	0.09	0.46	0.45	0.29	0.32	0.22 (1.5)	1.19 (0.90-1.56)
rs7004633	*MMP16*	8q21.3	G/A	0.03	0.37	0.60	0.06	0.36	0.57	0.21	0.25	0.24 (1.4)	1.20 (0.89-1.61)
rs7914558	*CNNM2*	10q24.32	G/A	0.23	0.53	0.24	0.29	0.49	0.22	0.49	0.53	0.23 (1.4)	1.16 (0.91-1.49)
rs11191580	*NT5C2*	10q24.33	T/C	0.49	0.42	0.09	0.50	0.42	0.07	0.70	0.72	0.55 (0.4)	1.09 (0.83-1.43)
rs12966547	*CCDC68*	18q21	G/A	0.15	0.44	0.41	0.17	0.44	0.39	0.37	0.39	0.54 (0.4)	1.09 (0.84-1.41)

*Abbreviations*: *Chr* Chromosome; *SCZ* patients with schizophrenia; *CON* healthy controls; +, risk allele; -, non-risk allele; *OR* odds ratio.

For alleles, the first allele is the risk allele. All risk alleles are represented based on the previous GWAS [7].

### Magnetic resonance imaging procedure

All magnetic resonance imaging (MRI) studies were performed on a 1.5 T GE Signa EXCITE system. A three-dimensional volumetric acquisition of a T1-weighted gradient echo sequence produced a gapless series of 124 sagittal sections using a spoiled gradient recalled acquisition in the steady state (SPGR) sequence (TE/TR, 4.2/12.6 ms; flip angle, 15°; acquisition matrix, 256 × 256; 1NEX, FOV, 24 × 24 cm; slice thickness, 1.4 mm). Subjects with MRI abnormalities, such as infarcts, hemorrhages or brain tumors, were screened out prior to including this study as part of routine clinical diagnosis and treatment. Therefore, there were no gross abnormalities in any of the subjects. Each image was visually examined to eliminate images with motion or metal artifacts, and the anterior commissure-posterior commissure line was adjusted. The MRI images were processed using the VBM8 toolbox in Statistical Parametric Mapping 8 (SPM8; http://www.fil.ion.ucl.ac.uk/spm/software/spm8/) running on MATLAB R2013a (MathWorks, Natick, MA, USA) according to the VBM8-Toolbox Manual (http://dbm.neuro.uni-jena.de/vbm8/VBM8-Manual.pdf). The T1 images were normalized and segmented into GM, white matter (WM) and cerebrospinal fluid (CSF) using the VBM8 toolbox with defaults for the extended options. The modulated non-linear only (i.e., with no affine component) option was selected to create volumetric GM partitions. Finally, the images were smoothed with an 8-mm full-width, half-maximum isotropic Gaussian kernel.

### Statistical analyses

In genetic association analysis, we performed power calculations using the Power Calculator for Two-Stage Association Studies (http://www.sph.umich.edu/csg/abecasis/CaTS/) [28]. The power estimate was based on an allele frequency of 0.53 at rs7914558, a prevalence of 0.01, an alpha level of 0.05, and assuming varying degrees of odds ratio using a multiplicative model. In brain morphological analyses, we performed power calculations using the G*Power Version 3.1.5 [29]. The power estimate was based on an alpha level of 0.05, a power of 0.80 and assuming varying degrees of effect size using *t* tests. Standardized effect sizes were calculated using Cohen’s *d* method (http://www.uccs.edu/faculty/lbecker).

Statistical analyses of the demographic variables were performed using PASW Statistics 18.0 software (SPSS Japan Inc., Tokyo, Japan). Based on the assumption that most of demographic variables, such as age and education years, were not fitted to a normality distribution with the Kolmogorov-Smirnov test (*p* < 0.05), differences in clinical characteristics between patients and controls or between genotypes were analyzed using the non-parametric Mann–Whitney *U*-test for continuous variables, such as age and years of education, and *χ*^*2*^ tests for categorical variables, such as gender and handedness, as shown in Additional file 1: Table S1-S3. The presence of HWE was examined using the *χ*^*2*^ test for goodness-of-fit via SNPAlyze V5.1.1 Pro software (DYNACOM, Yokohama, Japan). The allelic distributions of each SNP between patients and controls were analyzed using *χ*^*2*^ tests with the SNPAlyze software. The significance levels for HWE and other statistical tests were set at two-tailed *p*-values, *p* < 0.01 and *p* < 0.05, respectively.

We performed a comprehensive exploratory whole brain search using the SPM8 statistical tools to examine the effects of the diagnosis, the genotype and their interaction of each SNP on GM volume in patients with schizophrenia and healthy subjects. As two-way ANOVA can simultaneously investigate these effects only in one model, these effects on GM volume were statistically assessed using a full factorial model for a 2 × 2 ANOVA with diagnosis (cases and controls) and genotype status (major-allele homozygotes and minor-allele carriers) as independent variables in SPM8. Gray matter volumes are correlated with age, and gender and years of education differed significantly between the patient and the control groups (Additional file 1: Table S1). Therefore, age, gender and years of education were included as covariates of no interest into the analyses to control for confounding variables. We contrasted GM volumes between the diagnostic groups (smaller volume region in patients with schizophrenia compared with healthy subjects), the genotype groups (smaller or larger volume region in minor-allele carriers relative to major-allele homozygotes) or the genotype-diagnosis interaction. Non-sphericity estimation was used. We applied a voxel-level height threshold of *p* < 0.001 (uncorrected for multiple comparisons) and clusters of more than 100 contiguous voxels were considered for the exploratory VBM analyses. And then we applied family-wise error (*FWE*) correction for multiple testing to avoid type I errors at the whole brain level. Eventually, the significance level was set at *p* < 0.05 (*FWE*-_corrected_). To obtain a cluster as large as possible, we extracted relative GM volumes from nominal clusters in bilateral inferiror frontal gyri at the lenient uncorrected threshold of *p* < 0.001 and cluster sizes > 100. The extraction of these relative GM volumes were performed after including confounding factors such as age, sex and education years and modulated by total brain volumes in the VBM analyses. Anatomic localization was performed according to both the MNI coordinates and Talairach coordinates, which were obtained from M. Brett’s transformations (http://imaging.mrc-cbu.cam.ac.uk/imaging/MniTalairach) and presented as Talairach coordinates.

## Results

Our study size of 173 cases and 449 controls had sufficient power (>80%) to detect a genetic effect at odds ratio of 1.43 or greater for rs7914558 when the allele frequency was 0.53. Unfortunately, in our sample sizes, there was no allelic association with schizophrenia for any of the five SNPs [rs7914558 (*CNNM2*), rs10503253 (*CSMD1*), rs7004633 (*MMP16*), rs11191580 (*NT5C2*) and rs12966547 (*CCDC68*)] (*p* > 0.22, Table 1).

**Figure 1:**
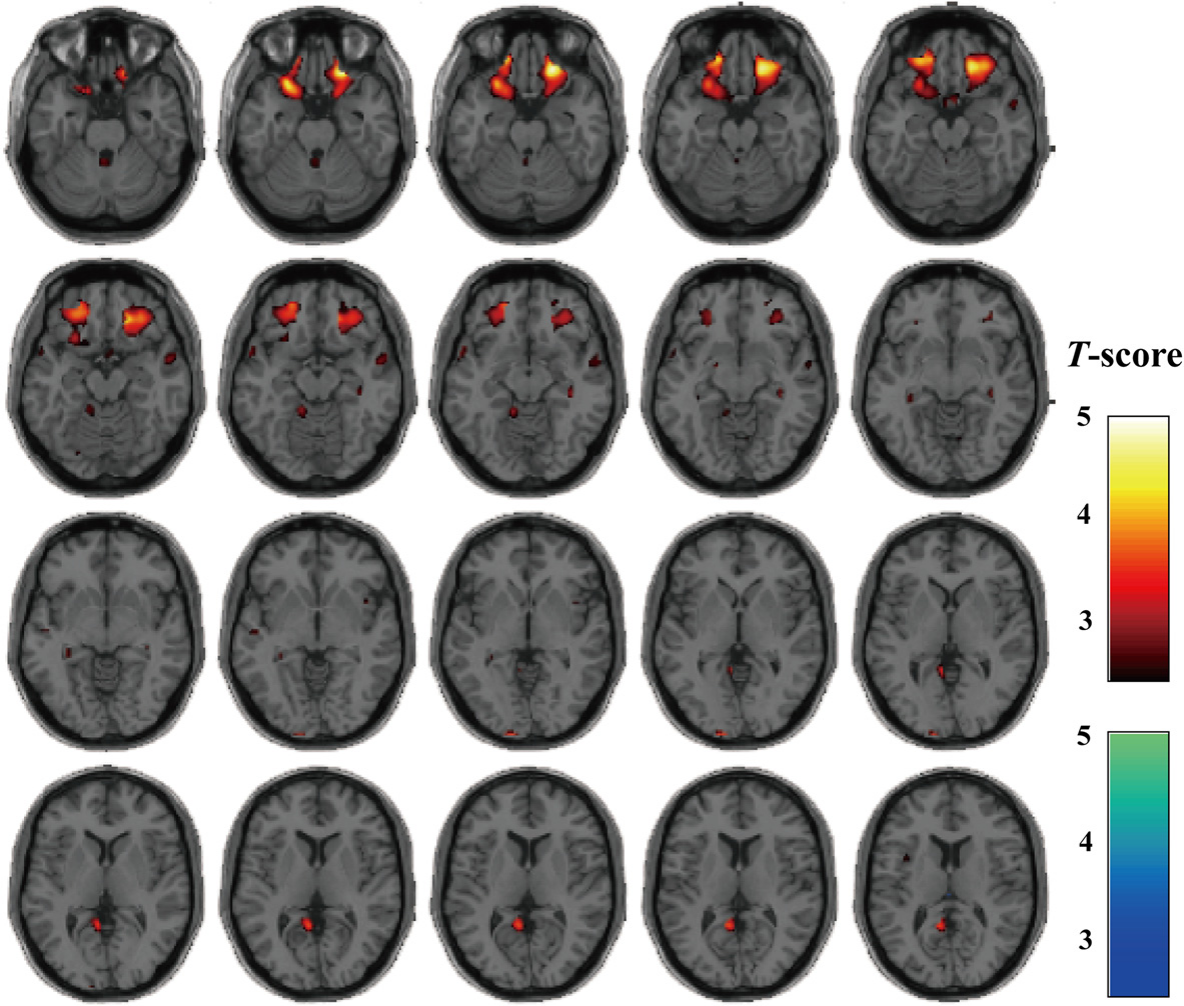
**Effects of the rs7914558 polymorphism at the *CNNM2 *gene on GM volumes.** There were effects of the risk-allele carriers of rs7914558 at *CNNM2* on decreased GM regions (red areas shown on the hot color map). There was no effect of the genotype on increased GM regions (blue area shown on the winter color map). Each color map shows the *t* values corresponding to the color in the figure.

**Figure 2:**
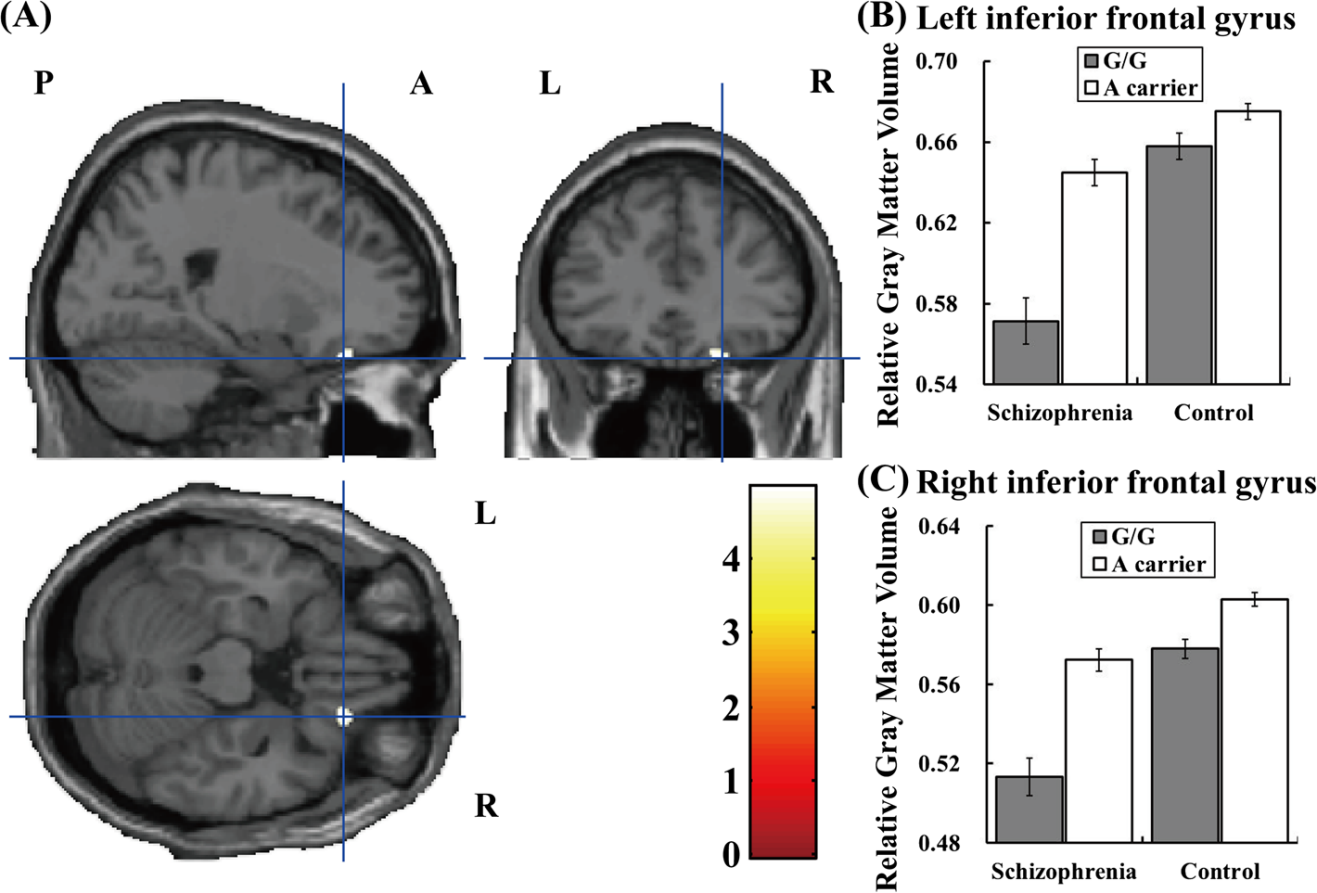
**The impacts of the *CNNM2 *genotype on GM volume of the bilateral inferior frontal gyri. (A)** Anatomical localizations are displayed on coronal, sagittal, and axial sections of a normal MRI spatially normalized into the Montreal Neurological Institute template (*p* <0.05). The most significant cluster of the genotype effect was in the right inferior frontal gyrus. The region is shown as a cross-hairline. The color bars show the *t* values corresponding to the color in the figure. **(B,C)** Each column shows the relative GM volumes extracted from a nominal cluster at _uncorrected_*p* < 0.001 and cluster size > 100 in the right inferior frontal gyrus (peak Talairach coordinates; 22, 31, -20) **(B)** and in the left inferior frontal gyrus (peak Talairach coordinates; -22, 18, -22) **(C)**. Error bars represent the standard error.

We investigated the effects of diagnosis (cases and controls), genotype (major-allele homozygotes and minor-allele carriers) and their interaction of five SNPs on GM volumes in a comprehensive exploratory VBM analysis. The effects of diagnosis between patients with schizophrenia and healthy subjects were found in all analyses of the present study (*p* < 0.05). Patients with schizophrenia showed smaller GM volumes compared with healthy subjects primarily in the frontal and temporal lobes, including the bilateral inferior frontal gyri, which was consistent with previous studies [14,30]. We found significant effects for the risk-allele homozygotes of rs7914558 on decreased GM volume in the bilateral inferior frontal gyri (right, *T* = 4.96, *p* = 0.0088; left, *T* = 4.66, *p* = 0.031), as shown in Table 2 and the regions based on the hot color map in Figure 1. To compare the effects of the genotype in both the patients with schizophrenia and healthy subjects, we extracted the means and SD for relative GM volumes from nominal clusters in bilateral inferior frontal gyri and the extracted GM volumes were shown in Figure 2 and Additional file 1: Table S2. The risk G-allele homozygotes of the *CNNM2* polymorphism had smaller GM volumes in the bilateral inferior frontal gyri compared to the non-risk allele carriers. The inferior frontal gyrus can be subdivided into three macroanatomical structures: the orbital (Brodmann area: BA47), opercular (BA44) and triangular (BA45) parts. In the present study, the peak GM regions affected by the *CNNM2* genotype existed in the orbital parts of the bilateral inferior frontal gyri. When the two genotype groups (G-allele homozygotes and A-allele carriers) were divided into three genotype groups (individuals with G/G genotype, G/A genotype and A/A genotype) and we performed an additional VBM analysis using a multiple regression model, the number of risk G-allele was significantly related to smaller GM volumes of the right inferior frontal gyrus (*T* = 4.67, *p* = 0.029) but not the left inferior frontal gyrus (*T* = 3.64, *p* = 0.70) in total subjects.

**Table 2:** **Effects of the *CNNM2 *** **genotype and genotype-diagnosis interaction on GM volumes**

**Brain regions**	**R/L**	**BA**	**CS**	***p*** **values (peak)**		**Talairach coordinates**		
				***T***	***FWE***	***x***	***y***	***z***
**Non-risk minor allele carrier > Risk major allele homozygote**								
Inferior frontal Gyrus	R	11/47	1306	4.96	**0.0088**	22	31	-20
Inferior frontal Gyrus	L	47	437	4.66	**0.031**	-22	18	-22
Middle frontal Gyrus	L	11	667	4.33	0.11	-24	38	-18
Posterior cingulate	L	29	248	3.73	0.61	-7	-48	11
**Non-risk minor allele carrier < Risk major allele homozygote**								
no suprathreshold clusters								
**Genotype-diagnosis interaction**								
Superior temporal Gyrus	L	22	598	4.23	0.16	-46	-20	1

*Abbreviations*: *R* right; *L* left; *BA* Brodmann area; *CS* Cluster size; *FWE* family-wise error.

All regions shown have nominal association at a voxel-level height threshold of _uncorrected_*p* < 0.001 and a minimum clusters extent of 100 voxels. Significant results (*FWE*-_corrected_*p* < 0.05) are shown in bold face.

As shown in Additional file 1: Table S1, patients with schizophrenia participated in this study were chronic and the symptoms were moderately stable. There was no difference in demographic information, such as duration of illness or PANSS scores, between risk G-allele homozygotes and non-risk A-allele carriers of rs7914558 (*p* > 0.20, Additional file 1: Table S3). However, to find whether there was potential clinical impact on our outcomes, we additionally investigated the genotype effect on GM volume including chlorpromazine equivalent of total antipsychotics (mg/day), duration of illness or PANSS scores as covariant for VBM analyses only in patients. The genotype effect on these regions did not change even after controlling for these factors, suggesting that there was no potential clinical impact on our outcomes.

In the exploratory VBM analyses, we also found nominal effects of the risk-allele homozygotes of rs7914558 on decreased GM volume in the left middle frontal gyrus and the left posterior cingulate and a nominal genotype-diagnosis interaction with the GM volume in the left superior temporal gyrus (_uncorrected_*p* < 0.001, Table 2). Additionally, we found several marginal effects of genotypes and genotype-diagnosis interactions of other SNPs on GM volumes in the exploratory analyses (_uncorrected_*p* < 0.001, Additional file 1: Table S4 and Figure S1-S4). However, these effects of genotypes and genotype-diagnosis interactions on GM regions did not survive after the *FWE-*correction for multiple tests at the whole brain level (*p* > 0.05).

## Discussion

To date, it remains unclear whether the genome-wide significant risk variants for schizophrenia in a mega-analysis of GWASs influenced GM volumes. This study is the first to identify the GM morphology associated with genome-wide risk variants using a comprehensive VBM technique*.* Of the five genetic variants investigated in this study, we found influences of the *CNNM2* genotype on the bilateral inferior frontal gyri at the whole brain level. GM volumes in the bilateral inferior frontal gyri, particularly the orbital region, in the risk G-allele homozygotes of *CNNM2* polymorphism were smaller than those observed in the non-risk allele carriers.

In genetic association analysis, our sample size had sufficient power (>80%) to detect a genetic effect with odds ratio of 1.43 or greater for rs7914558. However, previous large GWAS has reported the genetic effect with low odds ratio of 1.10 for the SNP [7]. To detect such a small genetic effect, our sample size had insufficient power (12%), and a large sample size of at least 3400 patients and 3400 controls is needed. On the other hand, in brain morphological analyses, our sample size had sufficient power (>80%) to detect a genotype effect on GM volumes at medium effect size (Cohen’s *d)* of 0.25 or greater. The observed effect sizes on the inferior frontal gyri were medium to large (0.29-1.00). These findings suggest that the genome-wide supported variant of schizophrenia had larger effect on GM volumes of the inferior frontal gyri than diagnostic status, and support that GM volumes abnormalities were prominent intermediate phenotypes bridging the gap between a susceptibility genetic variant and diagnostic categorization.

Rs7914558 is located in intron1 of the *CNNM2* gene (also known as *ACDP2*) on chromosome 10q24.32. The *CNNM2* gene spans 160.3 kb of genomic DNA and contains eight exons. This gene belongs to a member of the ancient conserved domain-containing protein family because the protein shares a domain conserved in a large number of species ranging from bacteria to human [31]. Members of this protein family contain a sequence motif that is present in the cyclin box, a cyclic nucleotide-monophosphate (cNMP)-binding domain. The *CNNM2* gene has a ubiquitous expression pattern in humans [31]. In particular, the level of *CNNM2* expression in the brain is moderate to high (http://www.ebi.ac.uk/gxa/experiment/E-MTAB-37/ENSG00000148842). However, whether the expression level of this gene in the brains of patients with schizophrenia is lower or higher than that in healthy subjects is unknown. The encoded protein CNNM2 plays an important role in magnesium homeostasis by modulating Mg2+ concentration. The *CNNM2* mRNA is upregulated when there is a deficiency of magnesium in the brain [32]. CNNM2 mediated Mg2 + -sensitive Na + currents were blocked by increased extracellular Mg2+ concentrations [33]. We assessed the effect of the rs7914558 genotype on *CNNM2* expression using bioinformatics data (http://www.sanger.ac.uk/resources/software/genevar/[34]) to examine whether the rs7914558 genotype might be an expression quantitative trait loci (eQTL). *In silico* analysis showed that the *CNNM2* gene expression of the high-risk G genotype of rs7914558 was significantly lower than that of the non-risk genotype in the combined lymphoblast-derived HapMap CEU and YRI samples (*r* = -.23, *t* = -2.59, *p* = 0.011). The low expression of this gene resulted increased Mg2+ levels. Increased extracellular Mg2+ concentrations caused a decrease in the activity of the glutamate *N*-methyl-D-aspartate (NMDA) receptor [35]. These findings suggest that the *CNNM2* gene may play an important role in the hypofunction of the NMDA receptor, which is implicated in the pathophysiology of schizophrenia.

The inferior frontal gyrus has a multifunctional role in human behavior, interpersonal interactions and communication [36]. The inferior frontal gyrus consists primarily of the heteromodal association neocortex, which is a major site of involvement in schizophrenia [37]. Several studies have reported that the relative GM in patients with schizophrenia was significantly reduced in the bilateral inferior frontal areas [38-40]. The inferior frontal gyrus can be subdivided into three macroanatomical parts: orbital, opercular and triangular. The orbital region is one of the major regions of the social brain that connects to the orbitofrontal cortex [41], while the opercular and triangular regions form Broca’s area, which is an important region for speech-language production [42]. We found that the *CNNM2* genotype affects brain volumes in the orbital regions of the inferior frontal gyri. Functionally, the orbital region is thought to be involved in the processing of empathy [36] and sentence comprehension [43,44] in the left hemisphere and decision-making cognition [41] and fine movement control [36] in the right hemisphere. Social functions are widely impaired in patients with schizophrenia [45-49]. It is still unclear whether and to what extent the effects of *CNNM2* polymorphism on GM structure observed here might be associated with an increased risk for schizophrenia. We suggest that the *CNNM2* variant may play a role in the social cognition and social functioning impairments noted in patients with schizophrenia through GM volumetric vulnerability of the orbital regions of the inferior frontal gyri. Further research is needed to investigate how a possible relationship between the *CNNM2* gene and hypofunction of the NMDA receptor would result in decreased GM volumes of the inferior frontal gyri.

There was no significant effect of the other four variants on any GM volumes. There are several possible reasons for the absence of an association. A false negative association cannot be excluded in our study because we applied a strict *FWE* correction for multiple comparisons at the whole brain level (*p* < 0.05). In the Additional file 1: Figure S1-S4, the regions shown at the more lenient uncorrected threshold of *p* < 0.001 may be helpful in further studies. Interestingly, many of the effects of these genome-wide significant variants at the lenient level involved decreased GM volumes, including the medial, middle and inferior fontal gyri. Reduced GM volumes in these regions have been repeatedly demonstrated in imaging studies of schizophrenia [14,30]. Another interpretation is that the effect of these variants was not sufficiently sensitive to the morphological vulnerability of the GM volumes. The effect of these variants may be preferable in identifying genotype-related vulnerability on other intermediate phenotypes, such as cognitive functions and personality traits. Therefore, further research is needed to confirm whether the effects of these variants could be related to the susceptibility of cognitive functioning.

There were several limitations to this study. Because a number of statistical analyses, including the effects of diagnosis, genotypes and their interaction on GM volumes, were performed, a correction for multiple testing should be considered. However, a consensus on how to correct such multiple testing on study combining brain imaging and genetics has not been reached in this research field. To control type I errors, we applied the strict *FWE* correction for all VBM analyses, while we did not perform any correction on the genetic modality. The existence of a false positive association cannot be excluded as an explanation for our results, although we were quite careful to match ethnicity and correct for multiple testing. Further investigations of other samples with much larger sample sizes and/or with different ethnicities and/or in relatives of those with schizophrenia are needed to confirm our findings. It is unclear whether our results are directly/indirectly linked to the *CNNM2* polymorphism rs7914558, to other polymorphisms in high LD with this variant or to interactions between this variant and other variants. To determine whether rs7914558 is the most strongly associated variant for schizophrenia and brain structure in this gene, an extensive search for other functional variants at this locus is needed. Additionally, as with other risk variants for schizophrenia, clarifying the biological role of this SNP through *in vitro* and *in vivo* studies is important to improve the understanding of the pathophysiology of schizophrenia.

## Conclusions

We found that a genome-wide supported variant of *CNNM2* could be associated with GM morphological vulnerability of the bilateral inferior frontal gyri. These results suggest that there may be possible deleterious effects of the risk G-allele at *CNNM2* in the inferior frontal gyri, which may, at least partially, represent the mechanism by which *CNNM2* increases the risk for schizophrenia. Further research will be needed to clarify the function of the risk *CNNM2* variant on the pathophysiology of schizophrenia.

## Abbreviations

GM: Gray matter; VBM: Voxel-based morphometry; GWAS: Genome-wide association study; SNP: Single-nucleotide polymorphism; CNNM2: Cyclin M2; QT: Quantitative traits; DSM-IV: Diagnostic and statistical manual of mental disorders fourth edition; SCID: Structured clinical interview for DSM-IV; HWE: Hardy-Weinberg equilibrium; LD: Linkage disequilibrium; MRI: Magnetic resonance imaging; FWE: Family-wise error.

## Competing interests

The authors declare that they have no competing interests.

## Authors’ contributions

RH supervised the entire project, collected the data, wrote the manuscript, was critically involved in the design, analysis and interpretation of the data and was responsible for performing the literature review. KO was critically involved in the collection and analysis of the data, and contributed to the editing of the final manuscript and contributed intellectually to the interpretation of the data. HY and SU took part in genotyping. HY, YY, MF, MF, YW, MI, HK and MT contribute with sample collection and gave comments to the manuscript. All authors contributed to and have approved the final manuscript.

## Supplementary Material

Additional file 1: Table S1 Demographic information for patients with schizophrenia and healthy subjects. **Table S2:** Effects of the rs7914558 genotype on extracted relative GM volumes of the bilateral inferior frontal gyri. **Table S3:** Demographic information for risk G-allele homozygotes and non-risk A-allele carriers of rs7914558. **Table S4:** Impacts of each genetic variant on GM volumes in the exploratory VBM analyses. **Figure S1:** Effects of the rs10503253 polymorphism (*CSMD1*) on GM volumes. **Figure S2:** Effects of the rs7004633 polymorphism (*MMP16*) on GM volumes. **Figure S3:** Effects of the rs11191580 polymorphism (*NT5C2*) on GM volumes. **Figure S4:** Effects of the rs12966547 polymorphism (*CCDC68*) on GM volumes. Click here for file

## References

[B1] Sullivan PF Kendler KS Neale MC Schizophrenia as a complex trait: evidence from a meta-analysis of twin studies Arch Gen Psychiatry 2003 60 1187 1192 10.1001/archpsyc.60.12.1187 14662550

[B2] Sun J Kuo PH Riley BP Kendler KS Zhao Z Candidate genes for schizophrenia: a survey of association studies and gene ranking Am J Med Genet B Neuropsychiatr Genet 2008 147B 1173 1181 10.1002/ajmg.b.30743 18361404

[B3] Stefansson H Ophoff RA Steinberg S Andreassen OA Cichon S Rujescu D Werge T Pietilainen OP Mors O Mortensen PB Sigurdsson E Gustafsson O Nyegaard M Tuulio-Henriksson A Ingason A Hansen T Suvisaari J Lonnqvist J Paunio T Borglum AD Hartmann A Fink-Jensen A Nordentoft M Hougaard D Norgaard-Pedersen B Bottcher Y Olesen J Breuer R Moller HJ Giegling I Common variants conferring risk of schizophrenia Nature 2009 460 744 747 19571808 10.1038/nature08186 PMC3077530

[B4] O’Donovan MC Craddock N Norton N Williams H Peirce T Moskvina V Nikolov I Hamshere M Carroll L Georgieva L Dwyer S Holmans P Marchini JL Spencer CC Howie B Leung HT Hartmann AM Moller HJ Morris DW Shi Y Feng G Hoffmann P Propping P Vasilescu C Maier W Rietschel M Zammit S Schumacher J Quinn EM Schulze TG Identification of loci associated with schizophrenia by genome-wide association and follow-up Nat Genet 2008 40 1053 1055 10.1038/ng.201 18677311

[B5] Rose EJ Donohoe G Brain vs behavior: an effect size comparison of neuroimaging and cognitive studies of genetic risk for schizophrenia Schizophr Bull 2013 39 518 526 10.1093/schbul/sbs056 22499782 PMC3627766

[B6] Ohi K Hashimoto R Yasuda Y Nemoto K Ohnishi T Fukumoto M Yamamori H Umeda-Yano S Okada T Iwase M Kazui H Takeda M Impact of the genome wide supported NRGN gene on anterior cingulate morphology in schizophrenia PLoS One 2012 7 e29780 10.1371/journal.pone.0029780 22253779 PMC3257237

[B7] Ripke S Sanders AR Kendler KS Levinson DF Sklar P Holmans PA Lin DY Duan J Ophoff RA Andreassen OA Scolnick E Cichon S St Clair D Corvin A Gurling H Werge T Rujescu D Blackwood DH Pato CN Malhotra AK Purcell S Dudbridge F Neale BM Rossin L Visscher PM Posthuma D Ruderfer DM Fanous A Stefansson H Steinberg S Genome-wide association study identifies five new schizophrenia loci Nat Genet 2011 43 969 976 10.1038/ng.940 21926974 PMC3303194

[B8] Whalley HC Papmeyer M Romaniuk L Sprooten E Johnstone EC Hall J Lawrie SM Evans KL Blumberg HP Sussmann JE McIntosh AM Impact of a microRNA MIR137 susceptibility variant on brain function in people at high genetic risk of schizophrenia or bipolar disorder Neuropsychopharmacology 2012 37 2720 2729 10.1038/npp.2012.137 22850735 PMC3473338

[B9] Meyer-Lindenberg A Weinberger DR Intermediate phenotypes and genetic mechanisms of psychiatric disorders Nat Rev Neurosci 2006 7 818 827 10.1038/nrn1993 16988657

[B10] Tan HY Callicott JH Weinberger DR Intermediate phenotypes in schizophrenia genetics redux: is it a no brainer? Mol Psychiatry 2008 13 233 238 10.1038/sj.mp.4002145 18285755

[B11] Potkin SG Turner JA Guffanti G Lakatos A Torri F Keator DB Macciardi F Genome-wide strategies for discovering genetic influences on cognition and cognitive disorders: methodological considerations Cogn Neuropsychiatry 2009 14 391 418 10.1080/13546800903059829 19634037 PMC3037334

[B12] Kaymaz N van Os J Heritability of structural brain traits an endophenotype approach to deconstruct schizophrenia Int Rev Neurobiol 2009 89 85 130 19900617 10.1016/S0074-7742(09)89005-3

[B13] Rijsdijsk FV Viding E De Brito S Forgiarini M Mechelli A Jones AP McCrory E Heritable variations in gray matter concentration as a potential endophenotype for psychopathic traits Arch Gen Psychiatry 2010 67 406 413 10.1001/archgenpsychiatry.2010.20 20368516

[B14] Chan RC Di X McAlonan GM Gong QY Brain anatomical abnormalities in high-risk individuals, first-episode, and chronic schizophrenia: an activation likelihood estimation meta-analysis of illness progression Schizophr Bull 2011 37 177 188 10.1093/schbul/sbp073 19633214 PMC3004195

[B15] Leung M Cheung C Yu K Yip B Sham P Li Q Chua S McAlonan G Gray matter in first-episode schizophrenia before and after antipsychotic drug treatment. Anatomical likelihood estimation meta-analyses with sample size weighting Schizophr Bull 2011 37 199 211 10.1093/schbul/sbp099 19759093 PMC3004197

[B16] Ren W Lui S Deng W Li F Li M Huang X Wang Y Li T Sweeney JA Gong Q Anatomical and functional brain abnormalities in drug-naive first-episode schizophrenia Am J Psychiatryin press 10.1176/appi.ajp.2013.12091148 23732942

[B17] Goldman AL Pezawas L Mattay VS Fischl B Verchinski BA Chen Q Weinberger DR Meyer-Lindenberg A Widespread reductions of cortical thickness in schizophrenia and spectrum disorders and evidence of heritability Arch Gen Psychiatry 2009 66 467 477 10.1001/archgenpsychiatry.2009.24 19414706 PMC2719488

[B18] Meyer-Lindenberg A Imaging genetics of schizophrenia Dialogues Clin Neurosci 2010 12 449 456 21319490 10.31887/DCNS.2010.12.4/amlindenberg PMC3181991

[B19] Hashimoto R Hashimoto H Shintani N Chiba S Hattori S Okada T Nakajima M Tanaka K Kawagishi N Nemoto K Mori T Ohnishi T Noguchi H Hori H Suzuki T Iwata N Ozaki N Nakabayashi T Saitoh O Kosuga A Tatsumi M Kamijima K Weinberger DR Kunugi H Baba A Pituitary adenylate cyclase-activating polypeptide is associated with schizophrenia Mol Psychiatry 2007 12 1026 1032 10.1038/sj.mp.4001982 17387318

[B20] Hashimoto R Hirata Y Asada T Yamashita F Nemoto K Mori T Moriguchi Y Kunugi H Arima K Ohnishi T Effect of the brain-derived neurotrophic factor and the apolipoprotein E polymorphisms on disease progression in preclinical Alzheimer’s disease Genes Brain Behav 2009 8 43 52 10.1111/j.1601-183X.2008.00440.x 18786162

[B21] Hashimoto R Moriguchi Y Yamashita F Mori T Nemoto K Okada T Hori H Noguchi H Kunugi H Ohnishi T Dose-dependent effect of the Val66Met polymorphism of the brain-derived neurotrophic factor gene on memory-related hippocampal activity Neurosci Res 2008 61 360 367 10.1016/j.neures.2008.04.003 18501457

[B22] Hashimoto R Numakawa T Ohnishi T Kumamaru E Yagasaki Y Ishimoto T Mori T Nemoto K Adachi N Izumi A Chiba S Noguchi H Suzuki T Iwata N Ozaki N Taguchi T Kamiya A Kosuga A Tatsumi M Kamijima K Weinberger DR Sawa A Kunugi H Impact of the DISC1 Ser704Cys polymorphism on risk for major depression, brain morphology and ERK signaling Hum Mol Genet 2006 15 3024 3033 10.1093/hmg/ddl244 16959794

[B23] Ohi K Hashimoto R Yasuda Y Fukumoto M Nemoto K Ohnishi T Yamamori H Takahashi H Iike N Kamino K Yoshida T Azechi M Ikezawa K Tanimukai H Tagami S Morihara T Okochi M Tanaka T Kudo T Iwase M Kazui H Takeda M The AKT1 gene is associated with attention and brain morphology in schizophrenia World J Biol Psychiatry 2013 14 100 113 10.3109/15622975.2011.591826 22150081

[B24] Ohnishi T Hashimoto R Mori T Nemoto K Moriguchi Y Iida H Noguchi H Nakabayashi T Hori H Ohmori M Tsukue R Anami K Hirabayashi N Harada S Arima K Saitoh O Kunugi H The association between the Val158Met polymorphism of the catechol-O-methyl transferase gene and morphological abnormalities of the brain in chronic schizophrenia Brain 2006 129 399 410 16330500 10.1093/brain/awh702

[B25] Hashimoto R Ohi K Yasuda Y Fukumoto M Iwase M Iike N Azechi M Ikezawa K Takaya M Takahashi H Yamamori H Okochi T Tanimukai H Tagami S Morihara T Okochi M Tanaka T Kudo T Kazui H Iwata N Takeda M The impact of a genome-wide supported psychosis variant in the ZNF804A gene on memory function in schizophrenia Am J Med Genet B Neuropsychiatr Genet 2010 153B 1459 1464 10.1002/ajmg.b.31123 20957649

[B26] Ohi K Hashimoto R Yasuda Y Kiribayashi M Iike N Yoshida T Azechi M Ikezawa K Takahashi H Morihara T Ishii R Tagami S Iwase M Okochi M Kamino K Kazui H Tanaka T Kudo T Takeda M TATA box-binding protein gene is associated with risk for schizophrenia, age at onset and prefrontal function Genes Brain Behav 2009 8 473 480 10.1111/j.1601-183X.2009.00497.x 19566714

[B27] Lindenmayer JP Bernstein-Hyman R Grochowski S A new five factor model of schizophrenia Psychiatr Q 1994 65 299 322 10.1007/BF02354306 7831416

[B28] Skol AD Scott LJ Abecasis GR Boehnke M Joint analysis is more efficient than replication-based analysis for two-stage genome-wide association studies Nat Genet 2006 38 209 213 10.1038/ng1706 16415888

[B29] Faul F Erdfelder E Lang AG Buchner A G*Power 3: a flexible statistical power analysis program for the social, behavioral, and biomedical sciences Behav Res Methods 2007 39 175 191 10.3758/BF03193146 17695343

[B30] Ellison-Wright I Bullmore E Anatomy of bipolar disorder and schizophrenia: a meta-analysis Schizophr Res 2010 117 1 12 10.1016/j.schres.2009.12.022 20071149

[B31] Wang CY Shi JD Yang P Kumar PG Li QZ Run QG Su YC Scott HS Kao KJ She JX Molecular cloning and characterization of a novel gene family of four ancient conserved domain proteins (ACDP) Gene 2003 306 37 44 12657465 10.1016/s0378-1119(02)01210-6

[B32] Goytain A Quamme GA Functional characterization of ACDP2 (ancient conserved domain protein), a divalent metal transporter Physiol Genomics 2005 22 382 389 10.1152/physiolgenomics.00058.2005 15899945

[B33] Stuiver M Lainez S Will C Terryn S Gunzel D Debaix H Sommer K Kopplin K Thumfart J Kampik NB Querfeld U Willnow TE Nemec V Wagner CA Hoenderop JG Devuyst O Knoers NV Bindels RJ Meij IC Muller D CNNM2, encoding a basolateral protein required for renal Mg2+ handling, is mutated in dominant hypomagnesemia Am J Hum Genet 2011 88 333 343 10.1016/j.ajhg.2011.02.005 21397062 PMC3059432

[B34] Stranger BE Nica AC Forrest MS Dimas A Bird CP Beazley C Ingle CE Dunning M Flicek P Koller D Montgomery S Tavare S Deloukas P Dermitzakis ET Population genomics of human gene expression Nat Genet 2007 39 1217 1224 10.1038/ng2142 17873874 PMC2683249

[B35] Kochlamazashvili G Bukalo O Senkov O Salmen B Gerardy-Schahn R Engel AK Schachner M Dityatev A Restoration of synaptic plasticity and learning in young and aged NCAM-deficient mice by enhancing neurotransmission mediated by GluN2A-containing NMDA receptors J Neurosci 2012 32 2263 2275 10.1523/JNEUROSCI.5103-11.2012 22396402 PMC6621799

[B36] Liakakis G Nickel J Seitz RJ Diversity of the inferior frontal gyrus–a meta-analysis of neuroimaging studies Behav Brain Res 2011 225 341 347 10.1016/j.bbr.2011.06.022 21729721

[B37] Ross CA Pearlson GD Schizophrenia, the heteromodal association neocortex and development: potential for a neurogenetic approach Trends Neurosci 1996 19 171 176 10.1016/S0166-2236(96)10022-9 8723199

[B38] Buchanan RW Vladar K Barta PE Pearlson GD Structural evaluation of the prefrontal cortex in schizophrenia Am J Psychiatry 1998 155 1049 1055 9699693 10.1176/ajp.155.8.1049

[B39] Yamasue H Iwanami A Hirayasu Y Yamada H Abe O Kuroki N Fukuda R Tsujii K Aoki S Ohtomo K Kato N Kasai K Localized volume reduction in prefrontal, temporolimbic, and paralimbic regions in schizophrenia: an MRI parcellation study Psychiatry Res 2004 131 195 207 10.1016/j.pscychresns.2004.05.004 15465289

[B40] Suzuki M Nohara S Hagino H Kurokawa K Yotsutsuji T Kawasaki Y Takahashi T Matsui M Watanabe N Seto H Kurachi M Regional changes in brain gray and white matter in patients with schizophrenia demonstrated with voxel-based analysis of MRI Schizophr Res 2002 55 41 54 10.1016/S0920-9964(01)00224-9 11955962

[B41] Rogers RD Owen AM Middleton HC Williams EJ Pickard JD Sahakian BJ Robbins TW Choosing between small, likely rewards and large, unlikely rewards activates inferior and orbital prefrontal cortex J Neurosci 1999 19 9029 9038 10516320 10.1523/JNEUROSCI.19-20-09029.1999 PMC6782753

[B42] Amunts K Schleicher A Burgel U Mohlberg H Uylings HB Zilles K Broca’s region revisited: cytoarchitecture and intersubject variability J Comp Neurol 1999 412 319 341 10.1002/(SICI)1096-9861(19990920)412:2<319::AID-CNE10>3.0.CO;2-7 10441759

[B43] Sakai KL Nauchi A Tatsuno Y Hirano K Muraishi Y Kimura M Bostwick M Yusa N Distinct roles of left inferior frontal regions that explain individual differences in second language acquisition Hum Brain Mapp 2009 30 2440 2452 10.1002/hbm.20681 19003956 PMC6870581

[B44] Sakai KL Language acquisition and brain development Science 2005 310 815 819 10.1126/science.1113530 16272114

[B45] Bragado-Jimenez MD Taylor PJ Empathy, schizophrenia and violence: a systematic review Schizophr Res 2012 141 83 90 10.1016/j.schres.2012.07.019 22917950

[B46] Sevy S Burdick KE Visweswaraiah H Abdelmessih S Lukin M Yechiam E Bechara A Iowa gambling task in schizophrenia: a review and new data in patients with schizophrenia and co-occurring cannabis use disorders Schizophr Res 2007 92 74 84 10.1016/j.schres.2007.01.005 17379482 PMC2039912

[B47] Billeke P Aboitiz F Social cognition in schizophrenia: from social stimuli processing to social engagement Front Psychiatry 2013 4 4 23444313 10.3389/fpsyt.2013.00004 PMC3580762

[B48] Kuperberg GR Kreher DA Ditman T What can event-related potentials tell us about language, and perhaps even thought, in schizophrenia? Int J Psychophysiol 2010 75 66 76 10.1016/j.ijpsycho.2009.09.005 19765622 PMC3136365

[B49] Vrtunski PB Simpson DM Weiss KM Davis GC Abnormalities of fine motor control in schizophrenia Psychiatry Res 1986 18 275 284 10.1016/0165-1781(86)90114-9 3749386

