# Supplementary material for: The impact of the genome-wide supported variant in the cyclin M2 gene on gray matter morphology in schizophrenia
Source: Behav Brain Funct. 2013 Oct 25;9:40. doi: 10.1186/1744-9081-9-40 (PMC3874599; doi:10.1186/1744-9081-9-40)
Supplement: Additional file 1: Table S1 — Demographic information for patients with schizophrenia and healthy subjects. Table S2: Effects of the rs7914558 genotype on extracted relative GM volumes of the bilateral inferior frontal gyri. Table S3: Demographic information for risk G-allele homozygotes and non-risk A-allele carriers of rs7914558. Table S4: Impacts of each genetic variant on GM volumes in the exploratory VBM analyses. Figure S1: Effects of the rs10503253 polymorphism (CSMD1) on GM volumes. Figure S2: Effects of the rs7004633 polymorphism (MMP16) on GM volumes. Figure S3: Effects of the rs11191580 polymorphism (NT5C2) on GM volumes. Figure S4: Effects of the rs12966547 polymorphism (CCDC68) on GM volumes. [file 1744-9081-9-40-S1.doc]

**Additional file 1**

**Table S1 Demographic information for patients with schizophrenia and healthy subjects**

|  | **Schizophrenia** | **Control** |  |
| --- | --- | --- | --- |
| **Variables** | **(*n* = 173)** | **(*n* = 449)** | ***p* values (*z*)** |
| Age (years) | 36.0 ± 12.3 | 35.4 ± 12.8 | 0.37 (0.89) |
| Gender (male/female) | 102/71 | 214/235 | **0.012 (6.38)^a^** |
| Education (years) | 14.1 ± 2.5 | 14.9 ± 2.2 | **<0.001 (-3.56)** |
| Estimated premorbid IQ | 100.9 ± 10.3 | 107.9 ± 8.0 | **<0.001 (-7.57)** |
| Handedness [rt. / other (lt. or bil.)] | 164/9 | 420/29 | 0.56 (0.34)^a^ |
| Total gray matter volumes | 665.5 ± 66.9 | 680.8 ± 66.6 | **0.016 (-2.41)** |
| CPZeq. (mg/day) | 558.4 ± 520.6 | - | - |
| Age at onset (years) | 24.7 ± 10.0 | - | - |
| Duration of illness (years) | 11.3 ± 9.5 | - | - |
| PANSS positive symptoms | 19.0 ± 6.0 | - | - |
| PANSS negative symptoms | 19.9 ± 6.5 | - | - |
| PANSS general psychopathology | 42.2 ± 11.8 | - | - |

PANSS, Positive and Negative Syndrome Scale; CPZ-eq., chlorpromazine equivalent of total antipsychotics. Means ± SD and *p* values are shown. Significant *p* values are shown as bold face. ^a^*χ^2^* test. Complete demographic information was not obtained for all subjects (estimated premorbid IQ in patients: *n* = 162, estimated premorbid IQ in controls: *n* = 448; PANSS: *n* = 171).

**Table S2 Effects of the rs7914558 genotype on extracted relative GM volumes of the bilateral inferior frontal gyri.**

|  | **Schizophrenia** | | |  | **Control** | | |  | **Diagnosis**  **effect** | **Genotype**  **effect** |
| --- | --- | --- | --- | --- | --- | --- | --- | --- | --- | --- |
|  | **G/G** | **A carriers** | ***p* values (*z*)** |  | **G/G** | **A carriers** | ***p* values (*z*)** |  | ***p* values (*z*)** | ***p* values (*z*)** |
|  | **(*n* = 40)** | **(*n* = 133)** | **Cohen's *d*** |  | **(*n* = 125)** | **(*n* = 313)** | **Cohen's *d*** |  | **Cohen's *d*** | **Cohen's *d*** |
| Right inferior frontal gyrus | 0.51 ± 0.06 | 0.57 ± 0.06 | **<0.001 (-4.77)** |  | 0.58 ± 0.06 | 0.60 ± 0.06 | **<0.001 (-3.65)** |  | **<0.001 (-6.42)** | **<0.001 (-4.87)** |
| (22, 31, -20)^a^ |  |  | 1.00 |  |  |  | 0.33 |  | 0.60 | 0.60 |
| Left inferior frontal gyrus | 0.57 ± 0.07 | 0.64 ± 0.07 | **<0.001 (-4.74)** |  | 0.66 ± 0.07 | 0.68 ± 0.07 | **0.024 (-2.26)** |  | **<0.001 (-6.21)** | **<0.001 (-3.87)** |
| (-22, 18, -22)^a^ |  |  | 1.00 |  |  |  | 0.29 |  | 0.57 | 0.39 |

^a^ peak Talairach coordinates. To obtain a cluster as large as possible, we extracted relative GM volumes from nominal clusters in bilateral inferiror frontal gyri at the lenient uncorrected threshold of *p*<0.001 and cluster sizes>100. The extraction of these relative GM volumes were performed after including confounding factors such as age, sex and education years and modulated by total brain volumes in the VBM analyses. Means ± SD, *p* values using Mann-Whitney *U*-test and Cohen’s *d* are shown.

**Table S3 Demographic information for risk G-allele homozygotes and non-risk A-allele carriers of rs7914558**

|  | **Schizophrenia** | | |  | **Control** | | |
| --- | --- | --- | --- | --- | --- | --- | --- |
|  | **G/G** | **A carriers** | ***p* values (*z*)** |  | **G/G** | **A carriers** | ***p* values (*z*)** |
| **Variables** | **(*n* = 40)** | **(*n* = 133)** |  |  | **(*n* = 125)** | **(*n* = 313)** |  |
| Age (years) | 38.3 ± 12.8 | 35.2 ± 12.1 | 0.20 (1.27) |  | 34.9 ± 12.0 | 35.4 ± 13.0 | 0.91 (-0.12) |
| Gender (male/female) | 26/14 | 76/57 | 0.38 (0.79)^a^ |  | 63/62 | 144/169 | 0.41 (0.69)^a^ |
| Education (years) | 14.3 ± 3.0 | 14.0 ± 2.3 | 0.55 (0.60) |  | 14.9 ± 2.2 | 15.0 ± 2.1 | 0.51 (-0.66) |
| Estimated premorbid IQ | 101.3 ± 11.0 | 100.8 ± 10.1 | 0.73 (0.35) |  | 108.0 ± 8.2 | 108.0 ± 8.0 | 0.48 (-0.70) |
| Handedness [rt./other(lt. or bil.)] | 38/2 | 126/7 | 0.95 (<0.01)^a^ |  | 120/5 | 290/23 | 0.20 (1.67)^a^ |
| Total gray matter volumes | 661.0 ± 79.2 | 666.8 ± 63.0 | 0.78 (-0.28) |  | 683.4 ± 64.8 | 679.7 ± 67.7 | 0.59 (0.54) |
| CPZeq. (mg/day) | 646.6 ± 577.0 | 531.8 ± 501.8 | 0.23 (1.21) |  | - | - | - |
| Age at onset (years) | 25.0 ± 10.4 | 24.6 ± 9.9 | 0.80 (0.25) |  | - | - | - |
| Duration of illness (years) | 13.4 ± 11.0 | 10.7 ± 8.9 | 0.23 (1.19) |  | - | - | - |
| PANSS positive symptoms | 19.7 ± 5.9 | 18.8 ± 6.1 | 0.51 (0.66) |  | - | - | - |
| PANSS negative symptoms | 20.5 ± 5.5 | 19.8 ± 6.7 | 0.44 (0.77) |  | - | - | - |
| PANSS general psychopathology | 42.5 ± 11.3 | 42.1± 12.0 | 0.88 (0.16) |  | - | - | - |

PANSS, Positive and Negative Syndrome Scale; CPZ-eq., chlorpromazine equivalent of total antipsychotics. Means ± SD and *p* values are shown. ^a^*χ^2^* test. Complete demographic information was not obtained for all subjects (estimated premorbid IQ in patients: G/G *n* = 39, A carriers *n* = 123, estimated premorbid IQ in controls: A carriers *n* = 312; PANSS: A carriers *n* = 131).

**Table S4 Impacts of each genetic variant on GM volumes in the exploratory VBM analyses**

|  |  |  |  |  | ***p* values (peak)** | | **Talairach coordinates** | | |
| --- | --- | --- | --- | --- | --- | --- | --- | --- | --- |
| **SNP IDs (Gene)** | **Brain regions** | **R/L** | **BA** | **CS** | ***T*** | ***FWE*** | ***x*** | ***y*** | ***z*** |
| **rs10503253 (*CSMD1*)** |  |  |  |  |  |  |  |  |  |
|  | **Non-risk major allele homozygote > Risk minor allele carrier** | | | | | | | | |
|  | Inferior Frontal Gyrus | L | 47 | 145 | 3.77 | 0.58 | -48 | 39 | -12 |
|  | Middle Frontal Gyrus | R | 47 | 142 | 3.70 | 0.65 | 52 | 39 | -6 |
|  | **Non-risk major allele homozygote < Risk minor allele carrier** | | | | | | | | |
|  | no suprathreshold clusters |  |  |  |  |  |  |  |  |
|  | **Genotype-Diagnosis interaction** | | | | | | | | |
|  | Parahippocampal Gyrus | R | 30 | 426 | 4.40 | 0.087 | 22 | -39 | 2 |
|  | Parahippocampal Gyrus | L | 30 | 130 | 3.40 | 0.92 | -16 | -38 | 5 |
| **rs7004633 (*MMP16*)** |  |  |  |  |  |  |  |  |  |
|  | **Non-risk major allele homozygote > Risk minor allele carrier** | | | | | | | | |
|  | Cuneus | L | 18 | 228 | 3.90 | 0.42 | -24 | -85 | 26 |
|  | Postcentral Gyrus | R | 3 | 102 | 3.78 | 0.56 | 19 | -36 | 65 |
|  | **Non-risk major allele homozygote < Risk minor allele carrier** | | | | | | | | |
|  | no suprathreshold clusters |  |  |  |  |  |  |  |  |
|  | **Genotype-Diagnosis interaction** | | | | | | | | |
|  | Middle Temporal Gyrus | L | 39 | 150 | 3.96 | 0.36 | -36 | -74 | 13 |
| **rs11191580 (*NT5C2*)** |  |  |  |  |  |  |  |  |  |
|  | **Non-risk minor allele carrier > Risk major allele homozygote** | | | | | | | | |
|  | No Suprathreshold Clusters |  |  |  |  |  |  |  |  |
|  | **Non-risk minor allele carrier < Risk major allele homozygote** | | | | | | | | |
|  | Cerebellar Posterior Lobe | L | NA | 604 | 4.38 | 0.09 | -18 | -87 | -17 |
|  | Cerebellar Posterior Lobe | L | NA | 941 | 4.22 | 0.17 | -21 | -78 | -44 |
|  | Cerebellar Posterior Lobe | R | NA | 455 | 4.07 | 0.26 | 6 | -71 | -23 |
|  | Cerebellar Posterior Lobe | R | NA | 277 | 3.46 | 0.89 | 25 | -53 | -14 |
|  | Cerebellar Posterior Lobe | R | NA | 202 | 3.46 | 0.89 | 18 | -85 | -36 |
|  | **Genotype-Diagnosis interaction** | | | | | | | | |
|  | Posterior Cingulate | L | 31 | 136 | 3.79 | 0.55 | -13 | -53 | 22 |
| **rs12966547 (*CCDC68*)** |  |  |  |  |  |  |  |  |  |
|  | **Non-risk major allele homozygote > Risk minor allele carrier** | | | | | | | | |
|  | No Suprathreshold Clusters |  |  |  |  |  |  |  |  |
|  | **Non-risk major allele homozygote < Risk minor allele carrier** | | | | | | | | |
|  | Inferior Frontal Gyrus | R | 13 | 113 | 3.35 | 0.94 | 31 | 7 | -13 |
|  | **Genotype-Diagnosis interaction** | | | | | | | | |
|  | Fusiform Gyrus | L | 20 | 221 | 3.70 | 0.64 | -36 | -8 | -22 |
|  | Anterior Cingulate | R | 32 | 122 | 3.55 | 0.80 | 10 | 17 | -12 |

R: right, L: left, CS: Cluster size, *FWE*: family-wise error. All regions shown have nominal association at a voxel-level height threshold of _uncorrected_ *p* < 0.001 and clusters of more than 100 contiguous voxels.

**Rs10503253 at *CSMD1*:** There were significant effects of the risk-allele carriers of rs10503253 on decreased GM volume in the left inferior frontal gyrus and the right middle frontal gyrus (red regions in Figure S1) and a significant genotype-diagnosis interaction on GM volume in the bilateral parahippocampal gyrus.

**Rs7004633 at *MMP16*:** There were significant effects of the risk-allele carriers of rs7004633 on decreased GM volume in the left cuneus and the right postcentral gyrus (red regions in Figure S2) and a significant genotype-diagnosis interaction on GM volume in the left middle temporal gyrus.

**Rs11191580 at *NT5C2*:** There were significant effects of the risk-allele homozygotes of rs11191580 on increased GM volume in the bilateral cerebellar posterior lobe (blue regions in Figure S3) and a significant genotype-diagnosis interaction on GM volume in the left posterior cingulate.

**Rs12966547 at *CCDC68*:** There were significant effects of the risk-allele carriers of rs12966547 on increased GM volume in the right inferior frontal gyrus (blue regions in Figure S4) and a significant genotype-diagnosis interaction on GM volume in the left fusiform gyrus and the right anterior cingulate.

There was no significant effect of the risk-allele carriers of rs10503253 or rs7004633 on increased GM volumes, or the risk-allele carriers of rs12966547 or rs11191580 on decreased GM volumes (_uncorrected_ *p* > 0.001).

**Figure S1** **Effects of the rs10503253 polymorphism (*CSMD1*) on GM volumes.**

There were effects of the risk-allele carriers of rs10503253 on the decreased GM regions (red areas are shown by the hot color map) and on increased GM regions (blue areas are shown by the winter color map). Each color map shows the *t* values corresponding to the color in the figure.

**Figure S2** **Effects of the rs7004633 polymorphism (*MMP16*) on GM volumes.**

There were effects of the risk-allele carriers of rs7004633 on the decreased GM regions (red areas are shown by the hot color map) and the increased GM regions (blue areas are shown by the winter color map). Each color map shows the *t* values corresponding to the color in the figure.

**Figure S3** **Effects of the rs11191580 polymorphism (*NT5C2*) on GM volumes.**

There were effects of the risk-allele homozygotes of rs11191580 on the decreased GM regions (red areas are shown by the hot color map) and on increased GM regions (blue areas are shown by the winter color map). Each color map shows the *t* values corresponding to the color in the figure.

**Figure S4** **Effects of the rs12966547 polymorphism (*CCDC68*) on GM volumes.**

There were effects of the risk-allele carriers of rs12966547 on the decreased GM regions (red areas are shown by the hot color map) and on increased GM regions (blue areas are shown by the winter color map). Each color map shows the *t* values corresponding to the color in the figure.
